# Arksey and O′Malleyʼs consultation exercise in scoping reviews: A critical review

**DOI:** 10.1111/jan.15265

**Published:** 2022-04-22

**Authors:** Niels Buus, Lene Nygaard, Lene Lauge Berring, Lisbeth Hybholt, Stine Lundstrøm Kamionka, Camilla Blach Rossen, Rikke Søndergaard, Anette Juel

**Affiliations:** ^1^ Faculty of Medicine, Nursing and Health Sciences, Monash Nursing and Midwifery Monash University Melbourne Victoria Australia; ^2^ Department of Regional Health Research University of Southern Denmark Odense Denmark; ^3^ Research Unit for Gynaecology and Obstetrics, Department of Clinical Research, University of Southern Denmark Odense Denmark; ^4^ Department of Gynaecology and Obstetrics Odense University Hospital Odense Denmark; ^5^ Psychiatric Research Unit, Mental Health Region Zealand, Denmark Slagelse Denmark; ^6^ Center for Relationships and De‐escalation, Mental Health Region Zealand Slagelse Denmark; ^7^ Mental Health Services East Mental Health Region Zealand Roskilde Denmark; ^8^ Research Unit, Child and Adolescent Mental Health Odense Denmark; ^9^ Department of Clinical Research University of Southern Denmark Odense Denmark; ^10^ Elective Surgery Center Silkeborg Regional Hospital Silkeborg Denmark; ^11^ Danish Research Institute for Suicide Prevention, Mental Health Centre Copenhagen Copenhagen Denmark

**Keywords:** community participation, health services research, methods, nursing, review

## Abstract

**Aims:**

To explore how consultation exercises were described in a convenience sample of recent scoping reviews.

**Design:**

Critical literature review.

**Data sources:**

We searched PsycINFO, Embase, CINAHL and PubMed in July 2020. Our inclusion criterion was a peer‐reviewed journal article reporting a scoping review in Danish, English, Norwegian or Swedish.

**Review methods:**

We identified a convenience sample of articles (*n* = 66) reporting a consultation exercise as part of a scoping review. The descriptions of the consultation were charted, summarized and critically discussed.

**Results:**

The current analysis showed no widely accepted consensus on how to approach and report a consultation exercise in the sample of scoping reviews. The reports of stakeholder consultation processes were often brief and general, and often there were no reports of the effects of the stakeholder consultation processes. Further, there was no discussion of the principal theoretical problems mixing stakeholder voices and review findings.

**Conclusion:**

The finding that conventional research ethics and research methods often were suspended could indicate that the stakeholder consultants were in a precarious position because of power imbalances between researchers and stakeholder consultants. We suggest that a consultation exercise should only be included when it genuinely invites participation and reports on the effect of alternative voices.

**Impact:**

Scoping reviews are common across a range of disciplines, but they often lack definitional and methodological clarity. In their influential approach to scoping studies, Arksey and OʼMalley introduced an optional ‘consultation exercise’, which has been heralded as a valuable tool that can be used to strengthen the process and outcome of a scoping study and to support the dissemination of the studyʼs findings and its implications. However, there is no clear outline on about how to operationalize consultations of stakeholders in scoping studies/reviews. This article includes recommendations for consultation exercises, including encouraging an aspirational move from ‘consultation’ to ‘participation’.

## 1 INTRODUCTION

Scoping reviews are common across a range of disciplines and more recently, guidelines for conducting scoping reviews (Peters et al., 2020) and reporting scoping reviews (Tricco et al., 2018) have been published. However, scoping reviews often lack definitional and methodological clarity (Colquhoun et al., 2014; Khalil et al., 2016; OʼBrien et al., 2016). In their widely cited paper, Arksey and OʼMalley (2005) outlined an approach to scoping studies that included an optional consultation exercise that could ‘enhance’ the results. In this paper, we will focus on the use of stakeholder consultations in scoping studies/reviews, and following a critical review of current consultation practices, we will recommend on how to design and report stakeholder consultations in ways that can substantially strengthen scoping study/review processes and outcomes.

## 2 BACKGROUND

For Arksey and OʼMalley (2005), a scoping study is a type of literature review that differs from systematic reviews by addressing relatively broad topics and not asking very specific research questions (p. 20). In their paper, they focused on scoping studies whose purpose was to identify gaps in the existing research literature and research activity. Like systematic reviews, the scoping study/review process was described as rigorous and transparent procedures that others ultimately should be able to replicate, while at the same time iterative and non‐linear. Arksey and O′Malleyʼs outline included five stages, from identifying the research question to collating, summarizing and reporting the results (pp. 22–28). The consultation exercise was an optional sixth stage that could ‘inform and validate findings from the main scoping review’ (p. 23).

With regards to the consultation exercise, Arksey and OʼMalley (2005) were influenced by Oliver (2001), who described opportunistic and heuristic ‘consultations’ (p. 175) as part of a systematic review process that challenged conventional research knowledge. As part of a workshop for health promotion practitioners, Oliver presented a systematic review of smoking cessation programs for pregnant women written by Lumley. The presentation of the review was not received well by the workshop participants who felt that too much had not been taken into account in the reviewʼs conclusions. Oliver and the workshop participants decided to send a letter describing their concerns to Lumley, who subsequently invited Oliver to participate in an update of the review. As part of updating the review, Oliver spoke to pregnant women about smoking through a ‘rapid appraisal exercise’ (2001, p. 173), which created a different, more holistic awareness about the programs and their impacts on smokers and their babies as well as an awareness of the shortcomings of the dominating health research paradigm. Oliver (2001) suggested that involving practitioners and consumers in research can influence both research processes and research knowledge. In line with Oliverʼs (2001) reflections, Arksey and OʼMalley (2005) argued that a consultation exercise can add references to a reviewʼs literature search and provide insights that would not necessarily be identified through the literature review itself.

It is important to notice that Arksey and OʼMalley (2005) described the design of a scoping ‘study’, not simply the design of a scoping ‘review’. Their study design was *in effect* a mixed‐methods design involving a literature study (the scoping review) and an interview study (the consultation exercise), and they argued that the consultation exercise could create ‘added value’ to both the literature study and the findings of the overall scoping study (p. 29). However, it remained unclear when and how to consult with stakeholders and how to integrate information from the consultation exercise with the review findings in the overall study.

In a subsequent methods discussed, Levac et al. (2010) addressed some of these issues and recommended that consultation should be a required part of scoping studies and have a clear purpose that could include sharing preliminary findings with stakeholders, validation of findings and informing future research. They suggested preliminary review findings as a fruitful outset for consultation and recommended that authors provided clear descriptions of the type of stakeholders that would be consulted, how data would be collected during the consultation exercise, and how consultation exercise data would be integrated into the overall study. Levac et al. (2010) emphasized consultation as an opportunity for ‘knowledge transfer’ with stakeholders in the field that could potentially strengthen dissemination of the findings (p. 7). In the final part of their discussion, Levac et al. (2010) problematized different terminology around scoping approaches, which we believe highlighted a central design issue: Using the term ‘scoping study’, as originally suggested by Arksey and OʼMalley (2005), indicates a mixed‐methods design where *findings* from a distinct literature review and findings from a distinct consultation exercise are integrated in an overall scoping study design, whereas ‘scoping review’ indicates a design, where *data* from a consultation exercise are merged into a scoping review process. Levac et al. (2010) suggested using Arksey and O′Malleyʼs (2005) terminology, which is more precise. However, this suggestion has not been taken up widely, and most ‘scoping studies’ are in fact referred to as ‘scoping reviews’. In the current paper, we will refer to ‘scoping studies’ when referring to Arksey and OʼMalleyʼs methods and terminology, and to ‘scoping review’ or ‘scoping study/review’ when referring to the wider field of literature reviewing.

Daudt et al. (2013) reflected on their use of Arksey and O′Malleyʼs (2005) framework and compared their own approach to the recommendations made by Levac et al. (2010). Most notably, Daudt et al. (2013) showed that the engagement of a large interprofessional team had been helpful for them, which *in effect* meant that they had had stakeholders involved throughout the whole process and that the previous distinction between research team and stakeholders was no longer clear. In line with Levac et al.’s (2010) recommendations, stakeholdersʼ contributions were described as useful for guiding future research as part of a knowledge translation process.

The stakeholder consultation in scoping studies/reviews has been examined as part of two ‘scoping reviews of scoping reviews’ (Pham et al., 2014; Tricco et al., 2016). Pham et al. (2014) reviewed a convenience sample of 344 scoping studies published between 1999 and 2012. Stakeholder consultation was reported in 38.9% of the sample. Stakeholders were commonly reported to assist in searching for literature (74.5%) and less commonly reported to partake in the interpretation of findings (30.7%) and providing commentary at the report writing stage (24.1%). Finally, 25.9% of the consultations were classified as ongoing throughout the study (Pham et al., 2014). Tricco et al. (2016) reviewed a convenience sample of 494 scoping reviews published between 1999 and 2014. Unfortunately, Tricco et al.’s (2016) findings cannot be compared directly to Pham et al.’s (2014), because they did not classify consultation activities in the same way. For instance, Tricco et al. (2016) regarded consultation exercises as ‘knowledge translation activities’ (p. 8), which did not include consultation during literature searching. They showed that 37% of the studies reported consulting topic experts and 27% reported consulting a librarian as part of their search strategies and that less than 10% of the sample reported an ‘end‐of‐grant’ knowledge translation (Tricco et al., 2016). We find these data and conclusions on the use of consultation exercises very general and therefore difficult to interpret. For instance, how should we consider studies that mention a stakeholder consultation without providing any evidence of the process and outcome?

## 3 THE REVIEW

### 3.1 Aim

In the literature reviewed above, there is a general consensus around the consultation exercise being a valuable tool that can be used to strengthen the process and outcome of a scoping study/review and to support the dissemination of the study/reviewʼs findings and its implications (Arksey & OʼMalley, 2005; Levac et al., 2010; Peters et al., 2020). However, there is no consensus about how to bring data from consultations of stakeholders into scoping studies and reviews. The aim of the current paper was to explore how consultation exercises are described in a convenience sample of recent scoping reviews and to provide recommendations for future consultation exercises in scoping studies and reviews.

### 3.2 Design

We were guided by three research questions: (1) At what stage of scoping studies were stakeholders consulted? (2) Who were the stakeholders and how were they consulted? and (3) How was information from stakeholders integrated into scoping studies?

### 3.3 Search methods

We used a relatively simple search strategy to identify a convenience (non‐probability) sample of articles reporting a consultation exercise as part of a scoping study/review. Our inclusion criterion was a peer‐reviewed journal article reporting a scoping study/review and published in Danish, English, Norwegian or Swedish. We searched PsycINFO, Embase, CINAHL and PubMed, because of these four databasesʼ collective scope. There were no limitations on dates of publication. The literature searches took place in July 2020.

### 3.4 Search outcome

The searches in PsycINFO, Embase and PubMed were identical and combined the free text searches, ‘scoping review’ AND (‘consultation*’ OR ‘knowledge translat*’) in ‘all text’. In CINAHL, we added the controlled heading, ‘scoping review’ to the search string. These searches identified 68 references in PsycINFO, 396 references in Embase, 211 references in CINAHL and 369 references in PubMed.

**FIGURE 1:**
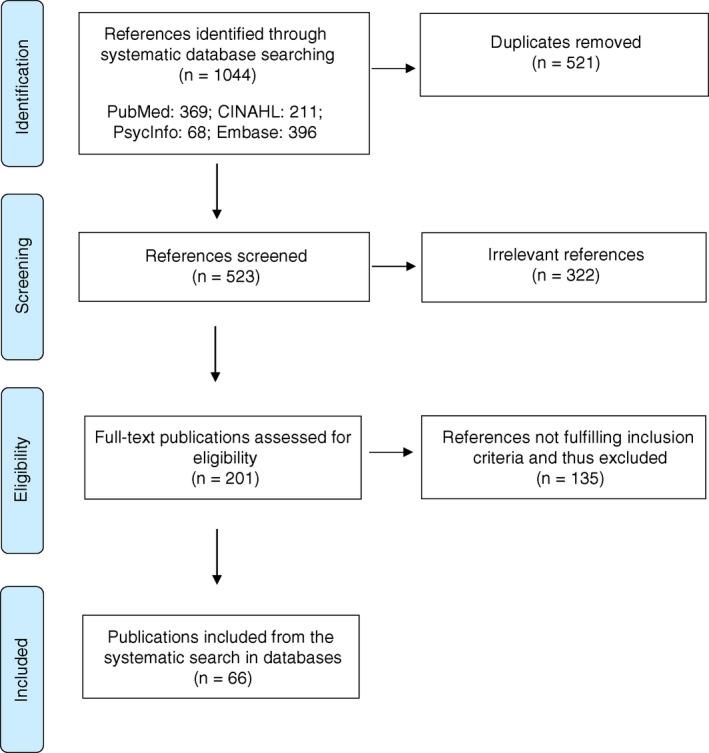
Flowchart illustrating the literature search process

We screened the studies using the following exclusion criteria: (1) Scoping studies that did not explicitly pay homage to the methods originally outlined by Arksey and OʼMalley (2005), (2) studies that did not include a mention of a consultation exercise, (3) mixed‐methods papers where details of the literature review were not sufficiently reported or the consultation exercise was reported in a different publication and (4) study protocols. To support the transparency and reliability of the study selection, we used the Covidence.org software to collaborate and document the search process. All references were reviewed by two independent reviewers, and if these two reviewers could not agree on exclusion or not, a third reviewer would be brought in and a consensus reached. A total of 66 studies were included. The search and inclusion processes of articles are summarized in Figure 1.

### 3.5 Data abstraction

First, each of the included articles was read by two reviewers who independently extracted and discussed data with regards to, (1) Where in the process stakeholders participated in the scoping review?, and (2) How their participation influenced the publication. Differences were discussed until a consensus was reached, which in some situations involved a third reviewer. Second, guided by our research questions, groups of two to three reviewers each focused on one of the three research questions by extracting and summarizing specific information from all the included papers. The groups made use of an additional reviewer, if they could not reach a consensus on how to read and interpret the description of the stakeholder consultations. This was needed because papers often lacked details on how consultation took place and what it resulted in. Third, data were tabulated and discussed by all authors.

## 4 RESULTS

Reports of stakeholder consultation processes were often brief and general, and often there were no reports of the effects of the stakeholder consultation processes.

Consultation took place in different stages of the review processes (see Table 1), with ‘consultation during literature searches’ (*n* = 24) and ‘consultation about interpreting findings’ (*n* = 45) as the most frequently reported, and ‘consultation during preparation phase’ (*n* = 11) and ‘consultation after completion of review’ (*n* = 11) as the less frequently reported. A small number of studies reported consultation throughout the whole review process (*n* = 8). Moreover, in the preparation stage, the most frequently reported use was ‘to generate research questions’; in the literature searching stage it was ‘to identify relevant studies’; in the findings stage it was ‘to get feedback/discussion of findings’, and in the after full review stage they were ‘to ensure user (practice or knowledge) relevance’, ‘to get feedback on review findings’ and ‘to inform further research’.

**TABLE 1:** The use and effects of using consultants at different stages in the scoping review processes

Stages in the scoping review process	Percentage of papers stating the use of consultation^a^	Percentage of papers describing evidence of any outcome of consultation^b^
Evidence of effect/outcome of the consultation exercise		80.3% (53/66)
Consultation during the preparation stage	16.7% (11/66)	54.5% (6/11)
Consultation during literature searching/review	36.4% (24/66)	54.2% (13/24)
Consultation about interpreting findings	68.2% (45/66)	73.3% (33/45)
Consultation after completion of review	16.7% (11/66)	72.7% (8/11)
Consultation throughout study	12.1% (8/66)	12.5% (1/8)

^a^ This includes any description of an effect disregarding the comprehensiveness of the description.

^b^ Percentage of papers where effects of consultation at all stages were described.

An effect of the consultation exercise was reported in 80.3% (53/66) of the studies. We categorized any description of an outcome related to the consultation exercise—no matter how modest—as an effect. Although this threshold resulted in a relatively high rate of observations of effect, we believed this was a more reliable approach than trying to define effect as more than a mere mention. Reports of outcomes of consultation varied greatly between the different stages. The outcome was reported proportionally more in ‘consultation about interpreting findings’ (73.3%, 33/45) and ‘consultation after completion of review’ (72.7%, 8/11), compared with ‘consultation during preparation stage’ (54.5%, 6/11) and ‘consultation during literature searches/review’ (54.2%, 13/24). The outcome from ‘consultation throughout study’ was reported proportionally the least (12.5%, 1/8), see Table 1.

The descriptions of consultations tend to be rather general. An example of this was Bakaki et al. (2018), who used stakeholder consultants in the preparation phase. They examined paediatric polypharmacy and periodically consulted a 10‐member stakeholder group during protocol development, data interpretation and reporting. The stakeholder consultants were experts in the content area and scoping reviews, not service user stakeholders. Bakaki et al. stated, ‘The team leader conceptualized the research questions and drafted the research protocol in consultation with the evidence synthesis expert and librarian. The rest of the team, including our stakeholders, reviewed, edited and approved the protocol before implementation. This iterative process ensured that the experts generated transdisciplinary research questions and approaches’ (Bakaki et al., 2018, p. 4). This description of the consultation exercise provided only a very general account of how the consultation took place and what changes were instigated. This was typical for the vast majority of the studiesʼ descriptions of the consultation exercise and only very few descriptions included a level of contextual details giving readers a sense of what took place. Finally, given Bakaki et al.’s role as experts, we assume that the stakeholdersʼ perspectives were relatively similar to the researchersʼ perspectives and therefore not providing an alternative ‘outsider’ perspective.

We could not identify a general pattern as to who qualified as a ‘stakeholder consultant’ or the purpose of ‘consultation’. Stakeholder consultants were described as having experience in the field in different ways and were, for instance, named ‘stakeholders’, ‘users by experience’, ‘experts’, ‘clinicians’, ‘topic population’, ‘academic experts’, ‘expert key informants’. The stakeholder consultants had different roles, such as professionals, researchers, consumer representatives and organizational decision‐makers and leaders. There were examples of papers, where the authors reported their own interdisciplinary team as providing multiple perspectives on the area of research and not involving genuinely alternative, external stakeholders.

The number of stakeholder consultants varied from 1 to >300 with a median number of 9. In 38% (25/66) of the papers, the number of stakeholder consultants was not provided. There was considerable variety in the methods used in the consultation exercises, related to whether the consultation was conducted individually, 21% (14/66), for instance interviews, and surveys, or in groups, 26% (17/66), for instance focus groups, workshops, public discussion, expert panels and advisory groups. Further, 14% (9/66) was mixed between individual and group‐based methods. In the remaining 39% (26/66) information about methods was not provided.

There was variation in terms of having a consultation once, 41% (27/66), or several times, 21% (14/66), over a period of time. In the remaining 38% (25/66) no information was provided about a single or multiple consultations.

There were seven articles, 11% (7/66), describing the use of several data sources, such as workshops combined with individual interviews and focus groups. 50% (33/66) used a single data source for example interviews, focus groups, workshops, email dialogue and questionnaires. The remaining 39% did not provide information about their use of single or multiple data sources (26/66). In eight papers, 12% (8/66), the methods of the consultation exercise were not explained and in approximately ¼ of papers, the exercise was just mentioned as a ‘consultation’, without any further elaboration.

**FIGURE 2:**
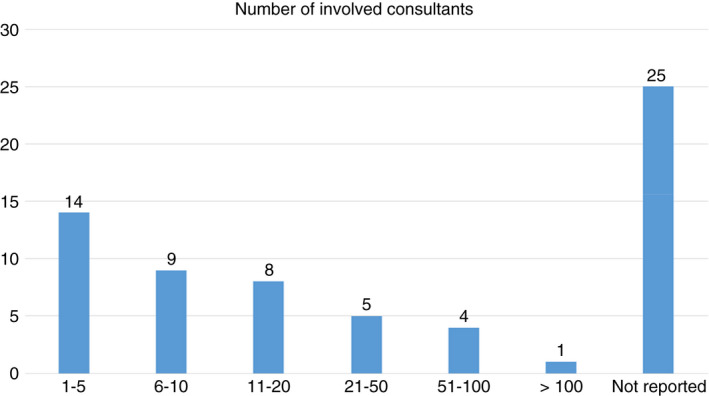
Number of involved consultants (*N* = 66)

In a scoping review of tools for assessing consultations by clinical ethics committees, a ten‐member ‘research team’ established an ‘expert team’ consisting of medical librarians, members of clinical ethics committees, academics, clinicians and educationalists (Yoon et al., 2020). The research team and the expert team collaboratively articulated the research question and designed the literature search. In the consultation exercise, ‘key stakeholders’ provided feedback on the preliminary review findings and they ‘were in agreement’ (Yoon et al., 2020). Neither the number of key stakeholders nor their roles or the character of the feedback process were provided in the paper. In line with Yoon et al.’s own rapport (2020), we decided to classify the stakeholder exercise as only taking place after the initial review despite *in effect* having user participation in the preparation phase and literature search phase (Figure 2).

Thirty‐three studies included findings from a consultation exercise that was not exclusively related to literature searching. Out of these, 39% (13/33) presented the findings from the stakeholder exercise as separate from the review and 61% (20/33) reported the findings from the consultation exercise as integrated with the review. None of the studies offered any epistemological reflections on the character and validity of the knowledge produced in these integrated reports.

The consultation exercises were rarely described as independent research studies in their own right: 23% (15/66) of the sample included considerations about ethics in relation to the consultation exercise data collection, e.g. formal approval from a research ethics committee or gathering consent from participating stakeholders, and 12% (8/66) offered reflections on the limitations of quality and validity of the consultation exercise, for example selection and number of stakeholders.

Oberlin et al. (2016) was an example of a paper reporting a scoping review where findings from a consultation exercise were integrated with the review findings. Oberlin et al. (2016) identified and reviewed relevant literature and conducted a consultation exercise that included interviews with six stakeholders. There was a brief description of each stakeholderʼs role and the focus of each interview, but no reports of methodological or research ethical reflections. Findings were reported thematically, and one theme had literature review findings mixed with findings from the interviews, for instance: ‘In health care, there is a growing amount of evidence that patients who are engaged, active participants in their own care, have better health outcomes and measurable cost savings.^[REF]^ The challenge is that patient engagement changes over time indicating a need for transplant centres to continue to cultivate and preserve relationships over the long term ([Name, date], consultative interview)’ (2016, p. 6). We are uncertain about how to interpret the epistemic claims made when findings are mixed and integrated in this way.

## 5 DISCUSSION

The current analysis showed no widely accepted consensus on how to approach and report a consultation exercise in the sample of scoping reviews. Furthermore, there was significant variation in the design of consultation exercises and stakeholders selected for the consultation exercises. There was no general agreement on who was qualified to be a stakeholder consultant.

Heavily influenced by Oliver (2001), Arksey and O′Malleyʼs (2005) optional consultation exercise introduced user consultation as part of a literature review method, which could be interpreted as a response to changing healthcare policies with increased pressure towards democratizing healthcare knowledge and practice through public participation (National Health and Medical Research Council, 2016). However, user participation is often criticized for being tokenistic with aspirational policies ahead of changing practices (Sangill, Buus, Hybholt, & Berring, 2019), and the current review showed that the practice and effects of consultation exercises were rarely reported in meaningful detail. While we cannot rule this out as a result of low‐prioritized or poor reporting, we believe this is indicative of a general tokenistic approach to user participation.

Also following Oliver (2001) and Arksey and OʼMalley (2005), a key contribution of the stakeholder consultants is to provide a perspective on a subject that challenges conventional perspectives and knowledge. Our observation that some studies included additional experts (on a research topic or literature searches), or even regarded their interdisciplinary team as ‘consultants’, can be regarded as being in direct conflict with the original intention of consulting stakeholders.

The finding that the scoping reviews rarely reported their consultation exercises in meaningful detail could indicate that the stakeholder consultants are in a precarious position because of power imbalances between researchers and the stakeholder consultants. *First*, consultation exercises were most often reported without sufficient reference to the research methodology. As a consultation exercise adds legitimacy to the findings of a scoping review, the trustworthiness of the contribution of the consultation should be transparent, rigorous and possible to assess by readers. This would, for instance, include methods to ensure balanced results through recruitment processes and analysis/interpretive processes. Stakeholders can ultimately be presented as ‘agreeing’ proponents of the results of a scoping review by researchers who have not rigorously researched and validly represented their situated perspectives.

*Second*, some studies considered the consultation as a formal data collection and reported obtaining approval from a research ethics committee to conduct the consultation exercise, and other studies reported gaining informed consent from stakeholder consultants. Other studies did not regard the consultation as a formal data collection and stated that ethical approval and informed consent were not applicable or even required and refrained from describing the rationale for this decision. When researchers refrain from adhering to ethical guidelines, stakeholder consultants are deprived of their rights as participants in research and are thus not guaranteed anonymity and do not hold the opportunity to withdraw their consent to participate. The consultation exercise also runs the risk of primarily fulfilling researchersʼ agendas (Tee & Lathlean, 2004), and could potentially violate the interests of stakeholder consultants. For instance, none of the reviewed scoping studies considered disagreement with stakeholder consultants and strategies to address situations where researchers and stakeholders have competing or even opposing interests. The consultation exercise becomes less of a partnership and comes across as research on stakeholder consultants rather than research with stakeholder consultants (Berring, Buus, & Hybholt, 2021). However, since consultation exercises can be characterized as a type of participatory research, reflections on ethical issues are highly important in that stakeholder consultants might be potentially vulnerable groups, for example patients or service users. When stakeholder consultants become co‐researchers, researchers are accountable for ensuring the protection and autonomy of stakeholder consultants (Tee & Lathlean, 2004).

Arksey and OʼMalley (2005) described a mixed‐methods approach to scoping studies where a scoping review was followed by an additional collection of data. They did not provide a position about the epistemic claims of such an approach, for instance, what types of knowledge are produced when stakeholder consultants reflect on review‐based knowledge? The review showed examples of this particular type of approach, which is aligned with a sequential mixed‐methods design where results from one discreet study (a scoping literature review) is followed up by a second discreet study (data collection in a consultation exercise) (Creswell & Plano Clark, 2007). However, our analysis also identified integrated designs where stakeholder consultantsʼ comments were juxtaposed or mixed with review findings. Such merging of expertise, data and knowledge fundamentally challenges modernist views on methods‐based rationality and scientific knowledge (Haraway, 1988). We often showed that the knowledge produced in the reviewed papers fruitful, but we are concerned about a general lack of researcher reflexivity in research processes, and a lack of theorizing the fuller consequences of the methodological and ethical implications of consultation exercises.

We interpret Arksey and O′Malleyʼs (2005) emphasis on validating review findings from the perspective of stakeholders as aligned with a pragmatic approach to mixed methods (Bourke Johnson & Onwuegbuzie, 2004). A pragmatic position considers the social good of knowledge production, and Biddle and Schafft (2015) referred to four constituent concepts of philosophy of knowledge ‘ontology, epistemology, axiology and methodology’ (Guba & Lincoln, 2005; Mertens, 2007) to discuss the failure of pragmatic mixed‐methods researchers to address axiology sufficiently. They do this by drawing on the ‘transformative paradigmʼs’ prioritized interest in politicizing research practices as a way to address power relationships and social injustice (Mertens, 2007). Biddle and Schafftʼs (2015) point can be used to highlight a shortcoming in Arksey and O′Malleyʼs (2005) approach to consultancy exercise with its aim to ‘inform and validate findings’ and Levac et al.’s (2010) subsequent aim to generate ‘knowledge transfer’; a stronger emphasis on social justice could transform scoping reviews by adding a reflexive stance on the politics of research. Validation of findings would include giving voice to historically disadvantaged and silenced voices; and knowledge transfer would include creating sustained social change.

As the analyses were based on a convenience sample of reported scoping reviews, we make no claims about the exact proportions of observations relative to ‘scoping reviews’ in general. The size and character of the sample allowed us to identify patterns, and our meticulous joint search and study selection practices were designed so that they would not add to skewing the sampleʼs characteristics. Further, as our general findings had substantial similarities with previous research (Pham et al., 2014; Tricco et al., 2016), we believe that our findings are relevant beyond the concrete analysis.

Mapping the reported consultation exercises was unexpectedly challenging because of limited and complex descriptions and we relied on several iterations of collaborative interpretations to be confident in the process. To provide transparency into the interpretations, we provide our raw mapping in the supplementary material.

## 6 CONCLUSION

Despite the fact that all of the articles in our sample were structured using Arksey and O′Malleyʼs (2005) six stages, the reported consultation exercises generally lacked descriptions of specific aims, designs and effects, which left the impression that simply stating that a consultation exercise had been done was a quality mark in itself rather than considering its potential value add.

Scoping reviewsʼ consultancy exercises need a re‐invigorating update, as they were largely tokenistic user participation practices. Considering the transformation of practices over time, we notice that Oliverʼs (2001) radical idea of bringing in alternative voices with an aim to challenge dominating research paradigms has had very little influence on current scoping review practices. We believe that the consultation exercises should be politicized to better address current social and health problems by genuinely activating community stakeholders and co‐develop strategies for sustained social change.

Furthermore, while Oliver and Arksey and OʼMalley (2005) focused on the benefits of Oliverʼs consultation with stakeholders, we argue that real change to the review process happened when Lumley invited Oliver to participate in the review update. Oliverʼs committed participation, not the pregnant womenʼs perspectives per se, allowed something genuinely new to happen in the review process. Influenced by Arnsteinʼs (1969) ladder of citizen participation, we argue that scoping reviews should climb the rungs of the ladder and include full participation by alternative stakeholders, rather than ‘just’ consulting them. However, for Arnstein, citizen participation was narrowly focused on power and control in decision‐making (Tritter & McCallum, 2006), and we believe that user ‘participation’ in literature reviewing can include other dimensions than a transfer of power, such as respectfully sharing different experiences and appropriately engaging with alternative, historically excluded, voices (as highlighted by Oliver).

Here, we close by recommending some principles for the development of reporting consultation exercises, which can provide transparency and align expectations to the role and contribution of the stakeholder consultants:

- Provide arguments as to how the selected stakeholders for the consultation exercise bring important different perspectives into the review process.
- Provide a comprehensive account of both the process and outcomes of the consultation exercise, including critical reflections on how the stakeholder perspectives from the consultation exercise challenged conventional perspectives.
- Treat the consultation exercise as research by:
  - following and reporting widely accepted approaches to research design and methodology, including data collection and data analysis.
  - following ethical principles for the protection of stakeholdersʼ interests and rights as research participants in research, such as giving informed consent.
- Treat the consultation exercise as part of a mixed‐methods study design, discuss the epistemological consequences of working with mixed methods, and report accordingly.

## CONFLICT OF INTEREST

The authors report no conflicts of interest.

### PEER REVIEW

The peer review history for this article is available at https://publons.com/publon/10.1111/jan.15265.

## Supporting information


Tables S1

## Data Availability

Our data can be made freely available as supplementary material.
